# Spatial structure facilitates evolutionary rescue by drug resistance

**DOI:** 10.1371/journal.pcbi.1012861

**Published:** 2025-04-03

**Authors:** Cecilia Fruet, Ella Linxia Müller, Claude Loverdo, Anne-Florence Bitbol

**Affiliations:** 1 Institute of Bioengineering, School of Life Sciences, ÉcolePolytechnique Fédérale de Lausanne (EPFL), Lausanne, Switzerland; 2 SIB SwissInstitute of Bioinformatics, Lausanne, Switzerland; 3 Sorbonne Université, CNRS,Institut de Biologie Paris-Seine (IBPS), Laboratoire Jean Perrin (LJP), Paris,France; University of Padova: Universita degli Studi di Padova, ITALY

## Abstract

Bacterial populations often have complex spatial structures, which can impact their evolution. Here, we study how spatial structure affects the evolution of antibiotic resistance in a bacterial population. We consider a minimal model of spatially structured populations where all demes (i.e., subpopulations) are identical and connected to each other by identical migration rates. We show that spatial structure can facilitate the survival of a bacterial population to antibiotic treatment, starting from a sensitive inoculum. Specifically, the bacterial population can be rescued if antibiotic resistant mutants appear and are present when drug is added, and spatial structure can impact the fate of these mutants and the probability that they are present. Indeed, the probability of fixation of neutral or deleterious mutations providing drug resistance is increased in smaller populations. This promotes local fixation of resistant mutants in the structured population, which facilitates evolutionary rescue by drug resistance in the rare mutation regime. Once the population is rescued by resistance, migrations allow resistant mutants to spread in all demes. Our main result that spatial structure facilitates evolutionary rescue by antibiotic resistance extends to more complex spatial structures, and to the case where there are resistant mutants in the inoculum.

## Author summary

Antibiotic resistance is a major challenge, since bacteria tend to adapt to the drugs they are subjected to. Understanding what conditions facilitate or hinder the appearance and spread of resistance in a bacterial population is thus of strong interest. Most natural microbial populations have complex spatial structures. This includes host-associated microbiota, such as the gut microbiota. Here, we show that spatial structure can facilitate the survival of a bacterial population to antibiotic treatment, by promoting the presence of resistant bacteria. Indeed, neutral or deleterious mutants giving resistance can take over small populations more easily than large ones, thanks to the increased importance of fluctuations in small populations. Resistant mutants can then spread to the whole structured population. Thus, population spatial structure can be a source of antibiotic treatment failure. This effect of spatial structure is generic and does not require environment heterogeneity.

## Introduction

Antibiotic resistance is a crucial challenge in public health [1,2]. Resistant bacteria emerge and spread through Darwinian evolution, driven by random mutations, genetic drift and natural selection. Mutations allow the emergence of strains adapted to challenging environments, including various antibiotic types [3]. These strains are selected when antibiotics are present in the environment. Critically, the evolution of antibiotic resistance can occur quickly, within days in specific conditions [4], while the development of a new antibiotic typically takes around ten years [5]. In this context, it is crucial to understand what conditions favor or hinder the development and spread of antibiotic resistance.

Bacterial populations often have complex spatial structures. For pathogenic bacteria, each patient constitutes a different environment, connected through transmission events. Within a patient, different organs and tissues represent spatial environments between which bacteria can migrate [6]. At a smaller scale, bacteria in the gut microbiota, which can be a reservoir of drug resistance, live in different spatial environments, namely the digesta, the inter-fold regions and the mucus, and some of them form biofilms [7–9], which are dense structures of cells enclosed in a polymer-based matrix [10,11]. Thus, it is important to understand how the spatial structure of microbial populations impacts the evolution and spread of resistance. This question has been explored in epidemiological models in the case of viruses [12,13] and of bacteria [14], and was recently addressed in controlled experiments, for specific antibiotic resistant bacteria [15], but is still relatively under-explored. Spatial structure can lead to small effective population sizes, and small population sizes were recently experimentally shown to substantially impact the evolution and spread of resistance [16,17]. More generally, the impact of spatial structure on the fate of mutants has been studied theoretically [18–36] and experimentally [37–41], but generally in constant environments. Furthermore, theoretical investigations have mainly focused on the fate of one mutant lineage, specifically on mutant fixation probability and fixation time.

Here, we ask how spatial structure impacts the overall fate of a bacterial population suddenly subjected to antibiotic treatment. Is the population eradicated by the drug, or does it survive by developing resistance? We address this question in a minimal model of population structure, composed of identical demes (i.e. subpopulations) connected to each other by identical migrations. This simple structure is known as the island model [18,19], or the clique or fully-connected graph [29,33,36]. We assume that the population initially only comprises drug-sensitive bacteria. Indeed, the initial diversity of a population of pathogenic bacteria in a host is often low, as many infections begin with a small infectious dose [42–46]. For simplicity, we assume that the environment is homogeneous, but note that environmental heterogeneities have a substantial effect on antibiotic resistance [47–49], and on evolutionary rescue in structured populations [50–52]. Specifically, we consider a perfect biostatic drug, which stops division of sensitive bacteria, but we also discuss the generalization of our results to other drug modes of action. When a well-mixed population is subjected to a treatment by a perfect biostatic drug (for a long enough time), the population gets extinct, except if resistant mutants are present when the drug is added [53–55]. The population can thus be rescued by resistance. We study the impact of population spatial structure on this process. We also analyze how the time when drug is added impacts the fate of a spatially structured population.

We show that spatial structure increases the probability that a bacterial population survives treatment by developing resistance through a neutral or deleterious mutation. Specifically, for a given time of addition of the antibiotic, the survival probability of the population increases when the migration rate between demes is decreased, and when the number of demes is increased while keeping the same total population size. We show that this is due to the local fixation of resistant mutants in one or several demes. We study the composition of the population versus time before the addition of drug, and find that it is strongly impacted by spatial structure. We discuss the parameter regimes where spatial structure facilitates rescue by resistance. We further study the time needed for resistant bacteria to colonize all demes after drug addition. Finally, we show that our main results regarding the impact of spatial structure on population survival to drug extend to more complex spatial structures, and to the case where resistant mutants are present in the inoculum.

## Models and methods

### Spatially structured populations.

**Fig 1:**
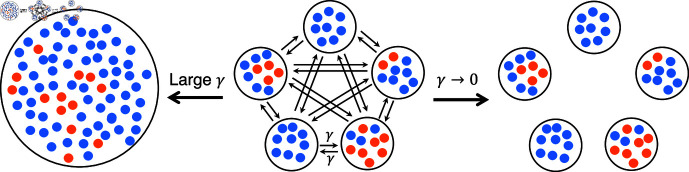
Minimal model of spatially structured populations. We consider a clique structure with one deme per node and a per capita migration rate *γ* (center). For large *γ*, it becomes a well-mixed population (left). Meanwhile, if *γ* → 0, it becomes a fully subdivided population. Blue and orange markers represent sensitive (S) and resistant (R) bacteria, respectively.

We aim to assess the impact of spatial structure on the establishment of antibiotic resistance, in a minimal model where spatial structure is as simple as possible. Thus, we consider a spatially structured bacterial population comprising *D* demes (i.e. subpopulations) on the nodes of a clique (i.e. a fully connected graph). This corresponds to the island population model [18]. Each deme has the same carrying capacity *K* (Fig 1, center). For comparison, we also consider a well-mixed population with the same total carrying capacity *DK* (Fig 1, left), and a fully subdivided population composed of *D* demes with carrying capacity *K* without migrations between them (Fig 1, right). We model migrations from one deme to another through a per capita migration rate *γ*, which is the same between all pairs of demes.

We will also briefly discuss extensions to more complex population structures on graphs [33,36].

### Bacterial division, death and resistance.

We consider two types of bacteria: sensitive (S) and resistant (R) ones. In the absence of antibiotics, the two types of bacteria have fitnesses $f_{S}$ and $f_{R}$, respectively, which set their maximal division rates (in the exponential phase). They have the same death rate *g*. Thus, we implement selection on birth. However, it is straightforward to generalize our model to selection on death. We assume that growth is logistic in each deme. The division rate of S (resp. R) individuals is $f_{S}\left(1-N∕K\right)$ (resp. $f_{R}\left(1-N∕K\right)$), where *N* is the current population size of the deme considered. When S bacteria divide, their offspring can mutate to R with probability *μ*. Here, we focus on the rare mutation regime, where *DKμ* ≪ 1.

We set $f_{S}=1$, implying that the time unit in our system is set by the maximum division rate of sensitive bacteria. Because antibiotic resistance often carries a fitness cost in the absence of drug [56,57], we write $f_{S}=1\;-\;\delta$, where *δ* represents the cost of resistance and satisfies 0 ≤ *δ* ≪ 1.

We do not model further mutations or backmutations – note that the rate of backmutations was estimated to be one order of magnitude smaller than the rate of compensatory mutations [58].

We consider an ideal biostatic drug that prevents division of the S bacteria, thus changing their fitness to $f_{S}=0$. We assume that R bacteria are not affected by the drug.

### Initial conditions and growth regime.

As we aim to model the appearance of resistance, we start with a population comprising only S bacteria. In practice, we initialize each deme (resp. the well-mixed population) with a number of S bacteria representing 10% of *K* (resp. of *DK*). Then, each deme (resp. the well-mixed population) quickly grows and reaches a steady-state size $N^{*}=K(1\;-\;g∕f_{S})$ (resp. $DN^{*}=DK(1-g∕f_{S})$) around which it fluctuates. We choose this initial condition because it is appropriate to model the start of an infection [59]. Note that since *DKμ* ≪ 1, it is very unlikely that mutations happen during this quick initial population growth phase, whose timescale is set by $f_{S}=1$ [54]. Our results are thus robust to the initialization, as long as it is performed only with S individuals.

We work in the regime where *K* ≫ 1 and $g\ll f_{S}=1$. Therefore, extinctions of demes are extremely slow in the absence of drug, and these events can be neglected here, as their timescales are much longer than all those we will discuss. (Specifically, the extinction of a deme with *K* = 100 starting with 10 S bacteria, with *g* = 0 . 1 and $f_{S}=1$, see Ref. [55], would take an average time $2.5\;\times \;10^{60}$ in the absence of drug.) Furthermore, in this regime, demes fluctuate weakly around their steady-state size in the absence of drug.

### Analytical and numerical methods.

We obtain analytical results from the Moran model [60] and from probability theory. Note that the Moran model assumes that population size is strictly fixed, which is not the case here. However, because we are in the regime where fluctuations around the steady-state size are small in the absence of drug, the Moran process provides good approximations (see Ref. [54]).

We perform stochastic simulations of the population evolution, using the Gillespie algorithm [61,62]. The details of the simulation framework are described in the S1 Appendix Sect 2.1.

## Results

### Spatial structure increases the probability that a bacterial population survives treatment

How likely is a spatially structured bacterial population to survive biostatic antibiotic treatment? Because our antibiotic prevents S bacteria from dividing while they have a nonzero death rate, a population is doomed to go extinct in the absence of R bacteria. However, if at least one R individual is present when drug is added, *rescue by resistance* can happen [53,54]. Here, we aim to assess the impact of spatial structure on rescue by resistance. To this end, we focus on the probability that the bacterial population survives antimicrobial addition in at least one of the demes. Indeed, R bacteria can then spread to the rest of the population via migrations, ensuring its overall survival.

**Fig 2:**
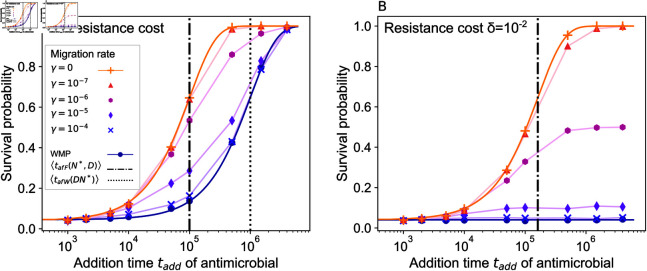
Survival probability of a structured bacterial population subjected to biostatic antimicrobial. The survival probability is shown versus the drug addition time. Panel A: Spatially structured populations composed of *D* = 10 demes with *K* = 100 are considered, with different per capita migration rates *γ*. Results for the well-mixed population with same total size (“WMP") are shown for reference. Markers report simulation results (each obtained from $10^{4}$ replicate simulations). The vertical dash-dotted line denotes the average time of appearance of a locally successful mutant in the fastest deme (Eq 2). The vertical dotted line represents the average appearance time of a successful mutant in a well-mixed population of size $DN^{*}$ (Eq 1). Solid lines for the fully subdivided (*γ* = 0) and well-mixed populations are analytical predictions from Eq 4. Thin solid lines connecting markers are guides for the eye. Panel B: Same as in Panel A, except that mutants have a cost of resistance $\delta =10^{-2}$. Parameter values: $f_{S}=1$ without drug, $f_{S}=0$ with drug, $f_{R}=1$ (Panel A), $f_{R}=0.99$ (Panel B), *g* = 0 . 1, $\mu =10^{-5}$.

Fig 2A shows the survival probability versus the time $t_{\text{add}}$ at which antimicrobial is added, for a spatially structured population with different values of the migration rate, in the case where resistant mutants carry no fitness cost, i.e. *δ* = 0. At the addition time $t_{\text{add}}$ of antimicrobial, the fitness of S bacteria switches from $f_{S}=1$ to $f_{S}=0$, while that of R bacteria remains the same. We observe that survival probability increases with $t_{\text{add}}$. Indeed, R mutants are more likely to appear and fix in the population, which is initially composed only of S bacteria, if there is more time before drug addition. Moreover, we find that survival probability is higher when migration rate *γ* is smaller. In S1 Appendix Sect 3.1, we further observe that subdividing a population with fixed total size into more numerous demes yields higher probabilities of survival.

Fig 2B shows the survival probability versus the drug addition time $t_{\text{add}}$ when resistant mutants have a small cost of resistance *δ* ≪ 1. We observe an even stronger impact of spatial structure on population survival than in the neutral case. Indeed, in the range of $t_{\text{add}}$ considered, the survival probability is roughly constant for the well-mixed population, while it strongly increases with $t_{\text{add}}$ for populations that are fully subdivided or have small migration rates. With the parameter choices of Fig 2B, resistant mutants are significantly deleterious in a well-mixed population (*DKδ* ≫ 1), while the impact of the cost is weaker within a deme (*Kδ* = 1). Accordingly, the well-mixed population behaves in a very different way in Fig 2A and 2B, while the fully subdivided one behaves similarly.

Note that the parameter values of Fig 2 were chosen to be in the rare mutation regime (*DKμ* ≪ 1, see “Models and methods”), and for computational tractability. The parameter regimes where spatial structure facilitates rescue by resistance will be determined in a dedicated section below, and the connection to realistic values will be made in the Discussion.

### Local fixation of R mutants promotes the survival of structured populations

#### Spatial structure allows local mutant fixation.

To rationalize our results on the impact of spatial structure on the ability of a microbial population to survive the addition of biostatic drug, we focus on rescue by resistance. Indeed, recall that we start from a sensitive inoculum. As mutations occur at division, R mutants arise with rate $N_{\text{tot}}^{*}\mu g$, where $N_{\text{tot}}^{*}$ is the steady-state total number of individuals in the population, *μ* the mutation probability upon division and *g* the death rate, which equals the division rate at steady state. All the populations we considered in Fig 2 have the same total steady-state size $N_{\text{tot}}^{*}=DK(1-g∕f_{S})$ in the absence of drug. Thus, the mutant supply is not impacted by spatial structure. However, the fate of mutants can be impacted by spatial structure.

Most R mutants that appear give rise to lineages that go extinct in the absence of drug, whatever the structure. Mutant lineages destined for extinction in the absence of drug can rescue the population if they are present when drug is added [54]. Indeed, once drug is added, S bacteria decay, and R bacteria can proliferate thanks to this reduced competition. However, this scenario remains unlikely in the rare mutation regime (*DKμ* ≪ 1), and is not strongly impacted by the value of $t_{\text{add}}$ or by population structure (see S1 Appendix Sect 1.1). Thus, it cannot explain the impact of spatial structure on rescue by resistance that we observe in Fig 2.

Let us now consider mutants that fix. Crucially, in spatially structured populations, fixation can occur locally within a deme. If migrations are rare enough, once R individuals have fixed in a deme, they survive there, provided that migrations are rare enough that we can neglect a subsequent migration and fixation of S bacteria in these R demes. Upon the addition of drug, these locally successful mutants rescue the population. R bacteria can then spread by migration to the whole population.

#### Timescales of appearance of mutants that locally fix.

To understand the impact of local mutant fixation on population survival, let us focus on successful mutants, i.e. those that give rise to a lineage that fixes. The average time $\left\langle t_{af\;W}(N^{*})\right\rangle$ of appearance (“*a*”) of a mutant that fixes (“*f*”) in a well-mixed population (“*W*”) of fixed size $N^{*}$ is given by [63,64]:


$$\begin{array}{ccc} \langle t_{af\;W}(N^{*})\rangle =\langle t_{\text{app}}(N^{*})\rangle \times \frac{1}{p_{\text{fix}}(N^{*})}=\frac{1}{N^{*}\mu g\;p_{\text{fix}}(N^{*})}\;, & & \end{array}$$ (1)

where $\left\langle t_{\text{app}}(N^{*})\rangle =1∕(N^{*}\mu g\right)$ is the average appearance time of a mutant, and $p_{\text{fix}}(N^{*})$ is its fixation probability, which can be expressed in the Moran model [65], see S1 Appendix Sect 1.2. Note that here, we are interested in the time until a successful R mutant appears [63,64], and not in its fixation time in a deme or in the full structured population [23,27,31].

In a structured population, the first mutant that fixes locally in one deme may rescue the population by its resistance. Neglecting the small fluctuations of demes around their steady-state size $N^{*}=K(1-g∕f_{S})$, the appearance of such a locally successful mutant can be approximated by a Poisson process with rate $1∕\langle t_{af\;W}(N^{*})\rangle$ (see Eq 1), and appearance time is exponentially distributed. Thus, the average appearance time of a locally successful mutant in the fastest (“*F*”) deme among *D* demes of steady-state size $N^{*}$ is (see S1 Appendix Sect 1.4 for details):


$$\begin{array}{ccc} \langle t_{af\;F}(N^{*},D)\rangle =\frac{\left\langle t_{af\;W}(N^{*})\right\rangle }{D}=\frac{1}{DN^{*}\mu g\;p_{\text{fix}}(N^{*})}\;. & & \end{array}$$ (2)

Crucially, a locally successful R mutant appears in the fastest deme of a structured population faster on average than a successful R mutant in a well-mixed population with the same total size. Indeed, Eqs 1 and 2 give


$$\begin{array}{ccc} \frac{\left\langle t_{af\;F}(N^{*},D)\right\rangle }{\left\langle t_{af\;W}(DN^{*})\right\rangle }=\frac{p_{\text{fix}}(DN^{*})}{p_{\text{fix}}(N^{*})}<1\;, & & \end{array}$$ (3)

for all resistance costs *δ* ≥ 0. This ratio is smaller than 1 because neutral or deleterious mutations fix more easily in small populations than in large ones, due to the increased effect of genetic drift, i.e. of fluctuations due to finite-size effects. For neutral R mutants (*δ* = 0), we have $p_{\text{fix}}(N^{*})=1∕N^{*}$, so the ratio in is equal to 1 ∕ *D*. Thus, a locally successful mutant appears in a structured population *D* times faster than a successful mutant in a well-mixed population with same total size. For R mutants with a nonzero cost of resistance *δ* > 0, the ratio in is even smaller, as mutant fixation is more strongly suppressed in larger population. See S1 Appendix Sect 3.2 for details.

Adding a biostatic antimicrobial (leading to $f_{S}=0$ and $f_{R}=1-\delta$ when drug is added) should thus have a different effect depending on the drug addition time $t_{\text{add}}$:

- If $t_{\text{add}}\ll \langle t_{af\;F}(N^{*},D)\rangle$, the bacterial population is likely to be eradicated, whatever its structure.
- If $\left\langle t_{af\;F}(N^{*},D)\rangle \ll t_{\text{add}}\ll \langle t_{af\;W}(DN^{*})\right\rangle$, it is likely that a locally successful mutant has appeared. This rescues a fully subdivided population (with no migrations). However, it is likely that no successful mutant has appeared in a well-mixed population yet. A well-mixed population is thus expected to go extinct.
- If $t_{\text{add}}\gg \langle t_{af\;W}(DN^{*})\rangle$, both a well-mixed and a fully subdivided population are expected to survive.

In Fig 2A, where neutral mutants are considered, we show the two timescales compared in Eq 3. We observe that the most substantial impact of structure on survival probabilities is observed when $\left\langle t_{af\;F}(N^{*},D)\rangle <t_{\text{add}}<\langle t_{af\;W}(DN^{*})\right\rangle$. This is fully in line with our theoretical analysis based on timescale comparisons. Furthermore, we observe that all populations survive if $t_{\text{add}}\gg \langle t_{af\;W}(DN^{*})\rangle$. Finally, if $t_{\text{add}}\ll \langle t_{af\;F}(N^{*},D)\rangle$, the population is eradicated in most cases. It can nevertheless be rescued by mutant lineages that were destined for extinction in the absence of drug. Thus, the probability of treatment survival is then given by the probability that non-successful mutants are present when the drug is added, see Eq S4 in the S1 Appendix.

In Fig 2B, we consider mutants with moderate fitness cost *δ* = 1 ∕ *K*. The average appearance time $\left\langle t_{af\;F}(N^{*},D)\right\rangle$ of a successful mutant in the fastest deme is shown (cf. and Eq S22) and is of the same order of magnitude as in the neutral case of Fig 2A. By contrast, these mutants are significantly deleterious in the well-mixed population since *DKδ* = *D* ≫ 1. Hence, $\left\langle t_{af\;W}(DN^{*})\right\rangle$ is not shown in Fig 2B, as it evaluates to $2.2\;\times \;10^{9}$, which is about $10^{4}$ times longer than $\left\langle t_{af\;F}(N^{*},D)\right\rangle$. The impact of spatial structure is even stronger in this case than for neutral mutants.

#### Analytical expression of the survival probability.

To go beyond our comparison of timescales, let us express the probability $p_{\text{surv}}(t_{\text{add}})$ that a population survives the addition of drug at time $t_{\text{add}}$, in a well-mixed population or in a fully subdivided population with no migrations. For this, we approximate deme size as constant, and we consider the two distinct mechanisms that can yield population survival, namely rescue by a (locally) successful mutant and rescue by the presence of the lineage of a mutant that was destined for extinction in the absence of drug. This leads to:


$$\begin{array}{ccc} p_{\text{surv}}(t_{\text{add}})=p_{\text{succ}}(t_{\text{add}})+\left[1-p_{\text{succ}}(t_{\text{add}})\right]p_{\text{pres}}\;, & & \end{array}$$ (4)

where $p_{\text{succ}}(t_{\text{add}})$ is the probability that a (locally) successful mutant has appeared by $t_{\text{add}}$, while $p_{\text{pres}}$ is the probability of presence of a lineage of R mutants destined to go extinct in the absence of drug. In the S1 Appendix, we calculate $p_{\text{pres}}$ in Sect 1.1 (see Eq S4) and $p_{\text{succ}}(t_{\text{add}})$ in Sect 1.6. The specific form of $p_{\text{succ}}(t_{\text{add}})$ is given by Eqs S28 and S29 for a well-mixed population, and by Eqs S31 and S32 for a fully subdivided population (with and without fitness cost, respectively).

Fig 2 shows that our simulation results are in very good agreement with the analytical predictions from Eq 4, both for the well-mixed population and for the fully subdivided population.

### Spatial structure impacts population composition before drug addition

To understand in more detail the role of spatial structure on resistance spread, let us study the system composition before drug is added. For simplicity, let us perform this analysis in the case of neutral R mutants, with the same parameter values as in Fig 2A.

**Fig 3:**
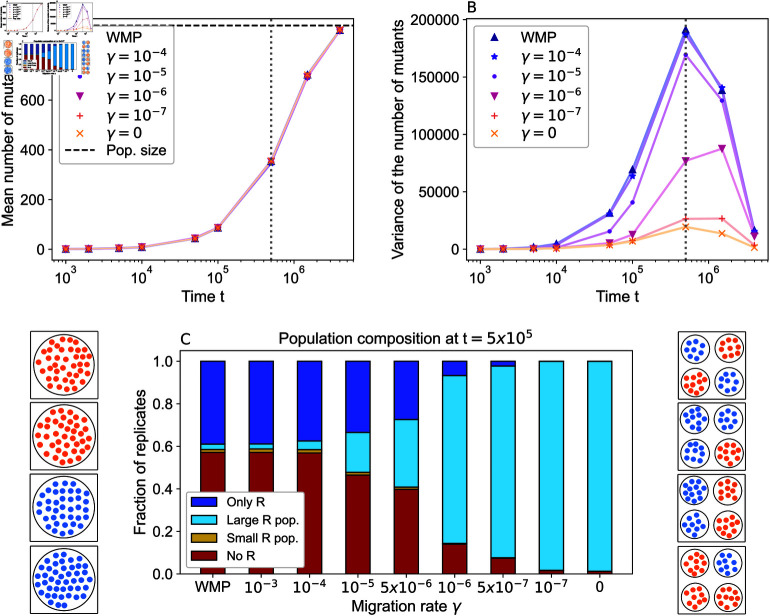
Impact of spatial structure on population composition in the absence of drug. Panel A: The mean across replicates of the total number of R mutants in the population is shown versus time for population structures differing by the migration rate *γ*, and for the well-mixed population with same total size (“WMP"). Horizontal dashed line: steady-state total population size $K\left(1-g∕f_{R}\right)$. Panel B: Variance across replicates in the total number of R mutants in the population, in the same conditions as in panel A. Vertical dotted line in panels A and B: time when largest variance was obtained in panel B ($t=5\times 10^{5}$). Panel C: Population composition at $t=5\times 10^{5}$, for different migration rates. We distinguish four cases. “Only R”: R bacteria have fixed in the whole population; “Large R population”: at least one deme comprises more than 9 R individuals, but the R type has not fixed in the whole population; “Small R population”: at least one deme comprises between 1 and 9 R bacteria, but no deme has more than 9 R; “No R”: there are no R bacteria. We distinguish “Large” and “Small R population” to account for the possibility of stochastic extinction of R lineages, which becomes negligible if  ≳ 10 R bacteria are present in a deme (see S1 Appendix Sect 3.4). Results are obtained from $10^{4}$ replicate simulations. Parameter values (in all simulations): *K* = 100, *D* = 10, $f_{S}=f_{R}=1$, *g* = 0 . 1, $\mu =10^{-5}$. Left and right of panel C: Schematics showing typical population states in four replicates (square boxes) for a well-mixed (left) and a fully subdivided population composed of *D* = 4 demes (right). Blue: S bacteria; orange: R bacteria.

Fig 3A shows the time evolution of the total number of R mutants present in a structured population, averaged over many stochastic simulation replicates. Whatever the migration rate, the average number of R individuals grows as mutants fix in some simulation replicates. This occurs either first locally for structured populations, or directly in the whole population for the well-mixed population (see S1 Appendix Sect 3.3). Despite this difference, the average over replicates is not impacted by population structure. Indeed, for neutral mutants, using and $p_{\text{fix}}(N^{*})=1∕N^{*}$ gives an average time $\left\langle t_{af\;W}(N^{*})\rangle =1∕(\mu g\right)$ for a successful mutant to appear in a well-mixed population. As this timescale is independent of population size, it governs the process of R mutants taking over each single deme but also a larger well-mixed population.

Fig 3B shows that the variance across replicates of the total number of R mutants in a population is strongly impacted by its structure. This is particularly true in the time interval between $\left\langle t_{af\;F}(N^{*},D)\right\rangle$ and $\left\langle t_{af\;W}(DN^{*})\right\rangle$ (see Fig 2A). This suggests that the impact of population structure on the variance of mutant number is driven by R mutant fixation. Fig 3B further shows that the variance of the number of mutants across replicates is larger when migrations are more frequent. In a well-mixed population, mutants fix in one step, while in a fully subdivided population, they fix separately in each deme (see S1 Appendix Sect 3.3). The large variance observed for the well-mixed population comes from the variability across replicates of the appearance time of a successful mutant. For the fully subdivided population, this is smoothed by lumping together *D* separate demes. Within each deme, the variance across replicates of the number of R mutants is not impacted by spatial structure, if R mutants are neutral, see S1 Appendix Sect 3.5.

In Fig 3C, we examine in more detail the population composition in the absence of drug, at the time when we obtained the largest variance in Fig 3B (vertical dotted line). We observe that in most replicates, the well-mixed population has either fixed resistance or does not have any R mutants. Thus, the number of mutants is either 0 or $(1-g∕f_{R})DK$, yielding the large variance seen in Fig 3B. Typical compositions of the well-mixed population are illustrated schematically on the left side of Fig 3C. As we partition the system spatially and reduce the migration rate, structured populations more frequently include some fully R demes and some fully S ones. Consequently, the fraction of replicates showing overall fixation or overall extinction decreases. This results in a smaller variance across replicates, as the number of mutants becomes more homogeneous across them. Typical compositions of the fully subdivided population are illustrated schematically on the right side of Fig 3C.

**Fig 4:**
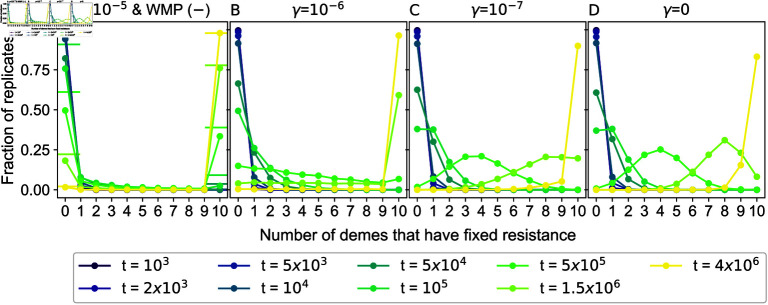
Number of demes that have fixed resistance before drug application. Histograms of the number of demes that have fixed resistance, computed across $10^{4}$ simulation replicates, are shown for different values of the elapsed time *t* (different colors), and of the migration rate *γ* (different panels). Specifically, for each *i* ∈ [ 0 , 10 ] , we show the fraction of realizations in which *i* demes have fixed resistance. The horizontal line markers in panel A show the results for a well-mixed population with same total size (“WMP"). Parameter values: *K* = 100, *D* = 10, $f_{S}=f_{R}=1$, *g* = 0 . 1, $\mu =10^{-5}$.

An important cause of the composition difference between structured and well-mixed populations is the possibility of local fixation of R mutants. What is the fixation dynamics at the deme level before we add the drug? In Fig 4, we report how many demes have fixed resistance for different migration rates and at different times, in the form of histograms computed over simulation replicates. Note that for the well-mixed population we report overall fixation. As expected, the number of demes where R mutants have fixed increases over time. Furthermore, we observe that the distribution of demes that have fixed resistance and its dynamics are strongly impacted by spatial structure. For large migration rates (panel A), at all times, most replicates have fixed resistance in either no deme or all demes. This is close to the large-*γ* limit (i.e. the well-mixed population), shown as horizontal lines in panel A. When the migration rate *γ* is decreased (panels B and C), the fraction of realizations featuring an intermediate number of demes that have fixed resistance increases at intermediate times. For the small migration rate $\gamma =10^{-7}$ (panel C), the distributions become close to those observed for *γ* = 0 (panel D). Thus, small migration rates result in a population with a transient strong heterogeneity across demes. This has a crucial impact on the outcome of drug treatment, and on the survival probability curves in Fig 2. Indeed, the biostatic drug does not affect R mutants, and the presence of at least one deme where R has fixed rescues the population when the drug is applied. Population composition is further shown versus time for different migration rates in the S1 Appendix Sect 3.6.

### Spatial structure facilitates rescue by resistance in broad parameter regimes

#### Mutation rate and population size.

As mentioned in “Models and methods”, we focus on the rare mutation regime *DKμ* ≪ 1. Outside of this regime, R mutants will usually be present in the population, and allow its rescue, independently of spatial structure. Another point where population size could have a role is that it takes longer for a lineage of R mutants destined to fix in a deme to actually fix there if the deme is larger. However, this does not affect our rescue scenario, because as long as the number of R mutants that are present when drug is added is sufficient for stochastic extinction not to occur after the addition of drug, the population will be rescued.

#### Cost of resistance.

For neutral resistant mutants (*δ* = 0), we saw that a locally successful mutant appears in a structured population *D* times faster than a successful mutant in a well-mixed population with same total steady-state size $DN^{*}$, where $N^{*}$ is the steady-state size of a deme. This leads to a substantial effect of structure on evolutionary rescue by drug resistance, see Fig 2A. For mutants with a substantial cost of resistance *δ* ≫ 1 ∕ *K*, the mutant fixation probability in a deme is $p_{\text{fix}}(N^{*})\approx \delta e^{-N^{*}\delta }$ (see S1 Appendix Sect 1.2), and thus the time until a successful mutant appears in a well-mixed population grows exponentially with $N^{*}$ (see Eq 1). The effect of spatial structure on rescue by resistance is then even stronger than for neutral mutants, but it exists at exponentially longer timescales (see S1 Appendix Sect 3.2). Finally, mutants with intermediate cost satisfying 1 ∕ ( *DK* ) ≪ *δ* ≪ 1 ∕ *K* are effectively neutral in demes of size $N^{*}$, but substantially deleterious in a well-mixed population of size $DN^{*}$. This regime yields a strong effect of spatial structure, which extends from the same timescales as in the neutral case to longer ones, as in Fig 2B (note that mutants are slightly more costly there: *δ* = 1 ∕ *K*). Thus, spatial structure facilitates evolutionary rescue by resistance for all costs *δ* ≥ 0, but the effect exists for extremely long timescales if *δ* ≫ 1 ∕ *K*. Because of this, we focus on small costs.

#### Migration rate.

So far, we mainly considered the extreme cases of the fully subdivided population and of the well-mixed population for our analytical reasoning. With the parameter values chosen in Fig 2, we observe that in practice, the transition between these two extreme cases occurs when the per capita migration rate is increased from $\gamma =10^{-7}$ to $\gamma =10^{-4}$. More generally, what is the relevant range of migration rates where spatial structure facilitates evolutionary rescue by drug resistance? Given the discussion above on the cost of resistance, let us focus on R mutants with cost satisfying *δ* ≪ 1 ∕ *K*. These mutants are neutral or effectively neutral within demes, and the fixation probability of an R (resp. S) individual in a deme of S (resp. R) bacteria is $p_{\text{fix}}(N^{*})=1∕N^{*}$.

We determined that spatial structure can facilitate evolutionary rescue by drug resistance because it fosters local fixation of R mutants. The demes that fix R mutants act as refugia for R mutants, and allow population survival when drug is added. For this to happen, local fixation of R mutants must be possible, without being perturbed by S bacteria migrating from other demes during the fixation process. The average fixation time of a neutral mutant in a well-mixed population of size $N^{*}$ is $\langle t_{\text{fix}}\rangle =(N^{*}\;-\;1)∕g$, where we used the Moran process result [65]. Meanwhile, the expected time until an S individual with a lineage destined for local fixation migrates into the deme where R is in the process of taking over is $\left\langle t_{\text{mig}}\rangle =1∕[N^{*}\gamma (D\;-\;1)p_{\text{fix}}(N^{*})]=1∕[\gamma (D\;-\;1)\right]$. Thus, for incoming migrations not to strongly perturb local fixations, we need $\left\langle t_{\text{fix}}\rangle \ll \langle t_{\text{mig}}\right\rangle$, i.e.


$$\begin{array}{ccc} \frac{\gamma }{g}\ll \frac{1}{\left(D\;-\;1)(N^{*}\;-\;1\right)}\;. & & \end{array}$$ (5)

Accordingly, in Fig 2A, spatial structure significantly impacts population survival when holds, i.e. when $\gamma \ll 10^{-4}$, and results become similar to those of a well-mixed population when $\gamma =10^{-4}$. Note that is less stringent than the rare migration regime [23,31,33], which requests a separation of timescales between migration and fixation processes. Here, we took into account the fact that only some migration events may strongly impact deme composition.

Once the fastest deme has fixed resistance, S bacteria may still migrate to that deme and fix there, leading to the extinction of R mutants. The expected time until this happens is again $\left\langle t_{\text{mig}}\rangle =1∕[\gamma (D\;-\;1)\right]$. Let us compare this to the average appearance time of a mutant that fixes in the fastest deme, given in Eq 2, i.e. $\left\langle t_{af\;F}(N^{*},D)\rangle =1∕(D\mu g\right)$ for effectively neutral mutants. If $\left\langle t_{\text{mig}}\rangle \gg \langle t_{af\;F}(N^{*},D)\right\rangle$, R mutants that locally fixed are expected to survive until other locally successful mutants appear. Then, provided that $t_{\text{add}}\gg \langle t_{af\;F}(N^{*},D)\rangle$, R mutants are present when drug is added, leading to rescue by resistance. Thus, spatial structure is expected to facilitate evolutionary rescue by resistance about as much as in a fully subdivided population if $\left\langle t_{\text{mig}}\rangle \gg \langle t_{af\;F}(N^{*},D)\right\rangle$, i.e.


$$\begin{array}{ccc} \frac{\gamma }{\mu g}\ll \frac{D}{D-1}\approx 1\;. & & \end{array}$$ (6)

Indeed, in Fig 2A, structured populations with migrations and fully subdivided ones have very similar survival probabilities when $\gamma \ll 10^{-6}$, i.e. when is satisfied. Survival probabilities intermediate between those of well-mixed populations and of fully subdivided populations are observed when is satisfied but not Eq 5. Note that while $\left\langle t_{\text{mig}}\right\rangle$ is the expected time until the next S individual destined for fixation migrates into the single R deme, R individuals from that deme may migrate to other demes. Taking this into account gives an expected extinction time of R mutants which is $\left\langle t_{\text{mig}}\right\rangle$ times a prefactor of order *log* ⁡  ( *D* ) , assuming rare migrations [66].

### R mutants readily colonize the whole population after drug addition

So far, we focused on the impact of spatial structure on the survival probability of the population upon drug addition. Let us now ask what happens after drug addition. With biostatic drug, S bacteria cannot divide or mutate, and their decay leaves some demes empty. If R mutants rescue a spatially structured population because they were present in at least one deme before drug addition, how fast do they then spread in all demes? This question has implications for disease recurrence, where after an initial transient alleviation of symptoms due to the drug-induced decay of S bacteria, the spread of R mutants could lead to a regrowth of the pathogenic bacteria population.

To address this question, let us first calculate the time $\left\langle t_{\text{c mig}}\right\rangle$ it takes for an R mutant to colonize an empty deme, starting from a population where resistance has fixed in *k* ≥ 1 demes out of *D*, while other demes are empty. Then, there are $N^{*}k$ mutant individuals, where $N^{*}$ is the steady-state deme size, which can migrate to each of the *D*–*k* empty demes at a per capita rate *γ*. However, once an R mutant arrives in an empty deme, its lineage may go stochastically extinct. This happens with probability $1\;-\;g∕f_{R}$ (see S1 Appendix Sect 1.1). Thus, we have:


$$\begin{array}{ccc} \langle t_{\text{c mig}}(k)\rangle =\frac{1}{\gamma N^{*}k(D-k)(1-g∕f_{R})}\;. & & \end{array}$$ (7)

**Fig 5:**
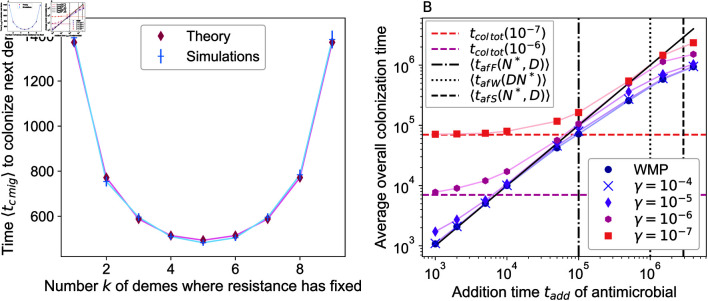
Colonization of the whole population by R mutants. Panel A: The time $\left\langle t_{\text{c mig}}\right\rangle$ to colonization of the next deme by R mutants through migrations after drug addition is shown versus the number *k* of demes where R mutants have already fixed. Migration rate: $\gamma =10^{-6}$. We compare simulation results from $5\;\times \;10^{3}$ realizations with the theoretical prediction from (“Theory”). Error bars represent 95% CI. Panel B: The average time to complete colonization by R mutants (i.e., when the number of R mutants first becomes at least equal to 0 . 9*K* in each separate deme) is shown versus the drug addition time $t_{\text{add}}$ for structured populations with different migration rates *γ*, and for the well-mixed population with same total size (“WMP"). Out of $10^{4}$ replicate simulations, we restrict to those where the population survived treatment. Horizontal lines: analytical prediction for $\left\langle t_{\text{c tot}}\right\rangle$ from Eq S36 for the two lowest *γ* values. Vertical lines: average local fixation time of R mutants in the fastest deme ($\left\langle t_{af\;F}(N^{*},D)\right\rangle$), in a well-mixed population ($\left\langle t_{af\;W}(DN^{*})\right\rangle$), and in the slowest deme ($\left\langle t_{af\;S}(N^{*},D)\right\rangle$). The black line *x* = *y* separates colonization occurring before drug is added (below the line) and after (above it). Confidence intervals are too small to be visible in this logarithmic scale. Parameter values for both panels: *K* = 100, *D* = 10, $f_{S}=f_{R}=1$, *g* = 0 . 1, $\mu =10^{-5}$.

In Fig 5A, we compare the analytical prediction in to our simulation results, obtaining a good agreement. The time $\left\langle t_{\text{c mig}}\right\rangle$ for R mutants to colonize the next deme features a minimum when half of the demes are mutant. Indeed, more mutant demes yield more mutants that may migrate, but fewer wild-type demes where they can fix, leading to a trade-off.

 neglects the presence of S bacteria in the population, which would affect the stochastic extinction of R lineages. This is acceptable provided that the extinction of S bacteria upon drug addition is fast enough. The decay time upon drug addition of a well-mixed population comprising $N^{*}$ S bacteria is $\tau_{S}=\left(1∕g\right)\sum_{i=1}^{N^{*}}1∕i$ [54]. For the migration rate considered in Fig 5A, $\tau_{S}$ is indeed negligible with respect to $\left\langle t_{\text{c mig}}\right\rangle$ (see S1 Appendix Sect 3.7). More generally, the condition $\tau_{S}\ll \langle t_{\text{c mig}}(1)\rangle$ yields $\gamma ∕g\ll 1∕[N^{*}(D\;-\;1)\sum_{i=1}^{N^{*}}1∕i]$, which is holds in most cases where spatial structure favors rescue by resistance (see Eq 5). The condition $\tau_{S}\ll \langle t_{\text{c mig}}(D∕2)\rangle$ is however slightly more demanding, as it yields $\gamma ∕g\ll 4∕[N^{*}D^{2}\sum_{i=1}^{N^{*}}1∕i]$.

So far, we focused on one step of the spread of R mutants. How long does it take in practice for R mutants to colonize the whole structured population after drug addition? First, if drug is added after a short time $t_{\text{add}}<\langle t_{af\;F}(N^{*},D)\rangle$, at most one deme in the population should comprise R mutants or have fixed them. Then, the average time $\left\langle t_{\text{c tot}}\right\rangle$ it takes for R mutants to colonize the whole population can be obtained by summing the step-by-step colonization times in Eq 7, see S1 Appendix Sect 3.7. In Fig 5B, we report the overall colonization time of the population by R mutants, defined as the time $t_{\text{add}}$ when drug is added plus the time needed for colonization after drug addition. We observe that for small addition times satisfying both $t_{\text{add}}\ll \langle t_{af\;F}(N^{*},D)\rangle$ and $t_{\text{add}}\ll \langle t_{\text{c tot}}\rangle$, the colonization time is indeed well-described by $\left\langle t_{\text{c tot}}\right\rangle$. It leads to a plateau whose value is governed by the migration rate. Note that in Fig 5B, this plateau is only observed for small migration rates ($\gamma ≲10^{-5}$) such that the condition $t_{\text{add}}\ll \langle t_{\text{c tot}}\rangle$ is satisfied for the smallest $t_{\text{add}}$ values considered. For larger values of $t_{\text{add}}$ and larger migration rates, the timescale $t_{\text{add}}$ dominates the overall colonization time of the population by R mutants, as $t_{\text{add}}>\langle t_{\text{c tot}}\rangle$, see Fig 5B. In these cases, mutant spread can be considered fast when drug is added. Finally, if $t_{\text{add}}$ is further increased, R mutants may fix in the whole population (i.e. colonize it) before drug addition. Thus, the overall colonization time becomes smaller than $t_{\text{add}}$ and tends toward a new plateau. Note that in this regime, colonization can occur before drug addition through migrations or new mutations, followed by fixations. The time it takes for a locally successful mutant to appear in the slowest deme can be expressed as $\langle t_{af\;S}(N^{*},D)\rangle =\langle t_{af\;W}(N^{*})\rangle \sum_{i=1}^{D}1∕i$ (see S1 Appendix Sect 1.3). It is beyond this time that we expect a plateau even for small migration rates. This is indeed what is observed in Fig 5B.

### Extensions to more complex spatial structures and heterogeneous inoculum

#### Rescue by resistance is also facilitated by spatial structures beyond the clique.

So far, we considered a minimal model of spatially structured population, known as the clique or the island model, where all demes are equivalent and connected to one another by identical migration rates (see “Models and methods”). We showed that spatial structure facilitates evolutionary rescue through the local fixation of resistant mutants in demes. This mechanism can also exist in different spatial structures, where demes are placed on graphs other than a clique.

We explore the impact of spatial structure on population survival upon drug addition in three structures with more reduced symmetry than the clique in the S1 Appendix Sect 4. First, we consider a two-dimensional square lattice with periodic boundary conditions. Second, we consider a star comprising a central deme connected to *D*–1 leaves, where all leaves are equivalent [33]. Third, we consider a line (or linear stepping-stone model) composed of *D* demes, where each deme is connected to two neighbors, except at the two ends [67]. In all cases, we choose the migration rates so that the total incoming migration rate to a deme is the same as in the clique, with the exception of the central deme in the star with asymmetric migrations, and of the end demes in the line with asymmetric migrations. This makes the re-invasion of an R deme by S bacteria as similar as possible across structures, but not fully. Our simulation results in the S1 Appendix Sect 4 show that all these structured populations have the same probability to survive treatment if $t_{\text{add}}<\langle t_{af\;F}(N^{*},D)\rangle$, i.e., in cases where usually not more than one deme has fixed resistance before drug addition. For larger values of drug addition time $t_{\text{add}}$, our results for the star depend on migration asymmetry and significantly differ from those obtained for other structures, although the difference remains minor. We interpret this difference as arising from S individuals possibly re-invading demes where R mutants have fixed, which remains structure-dependent.

Overall, our main result that spatial structure increases survival probability upon drug addition is qualitatively robust to changing the graph on which demes are placed, as it largely depends on local fixation of R mutants. However, migration patterns impact the re-invasion of R demes by S bacteria, and because of this, the conditions on migration rate we identified in Eqs 5 and 6 for the clique will differ for other graphs. The spread of R mutants to other demes by migration before drug addition, and the colonization of the graph by R mutants after drug is added, will also be affected by graph structure.

#### Inoculum that contains R mutants.

So far, we focused on a sensitive inoculum and on resistant individuals appearing through mutations (see “Models and methods”). However, resistant mutants can be present in the inoculum. This can be promoted e.g. by the presence of antibiotic in other hosts or in the environment. Furthermore, spatially structured populations starting with a fraction of R mutants in each deme were recently considered in *in vitro* experiments and modeling in Ref. [41]. To connect to these cases, we consider the initial condition where some mutants are already present in each deme of a clique, see S1 Appendix Sect 5. We observe that in this case too, spatial structure increases the survival probability of the population. Indeed, mutant extinction in all demes is less likely than mutant extinction in a well-mixed population.

## Discussion

Here, we showed that spatial structure can facilitate the survival of a bacterial population to antibiotic treatment, starting from a sensitive inoculum. Indeed, the bacterial population can be rescued if antibiotic resistant mutants are present when drug is added. While the emergence of resistant bacteria by random mutations only depends on total population size and not on spatial structure, their fate can be affected by spatial structure. If the mutation that provides resistance is neutral or deleterious, which is usually the case, its probability of fixation is increased in smaller populations. This leads to local fixation of resistant mutants in demes, which then constitute refugia of resistant mutants and allow the population to survive when drug is added. Because of this, spatial structure facilitates evolutionary rescue by drug resistance. The survival probability of the population increases when the migration rate between demes is decreased, and when the number of demes is increased. We showed that this effect exists in the rare mutation regime, for relatively rare to rare migration rates, and we quantified these conditions. While it holds for all costs of resistance, the timescales involved become very long for substantial costs. Thus, we mainly focused on neutral resistant mutants and those with moderate costs. After drug is added and the population is rescued by resistance, migrations allow resistant mutants to colonize all demes. While we considered a minimal model where all demes are equivalent and connected to each other with identical migration rates, bacterial populations can have diverse spatial structures. We showed that our key finding that spatial structure facilitates rescue by resistance still holds for more complex spatial structures, and when resistant mutants are present in the inoculum, as it relies on local fixation.

The effect of spatial structure we evidenced here is due to stochastic fixation of neutral mutants. Stochastic effects often give rise to original behaviors of small and structured populations. In particular, inoculum size impacts the survival of bacterial population in the presence of antibiotic [16,17], and spatial partitioning impacts the efficiency of antibiotic resistance by production of the beta-lactamase enzyme [15]. However, the effect evidenced here is different, as it relies on the stochasticity in the fate of a mutant, and not in the stochasticity in the inoculum size.

Because local fixation of neutral resistant mutants is instrumental to our effect, it involves rather long timescales. For neutral resistant mutants, the average appearance time $\left\langle t_{af\;F}\rangle =1∕(D\mu g\right)$ of a locally successful mutant in the fastest deme is of particular relevance. As it scales as the inverse mutation probability per individual and per generation 1 ∕ *μ*, it can be quite long, even considering that there are often several different mutational targets that give rise to resistance, leading to a larger effective *μ*. However, this timescale is inversely proportional to the number *D* of demes, meaning that it can potentially become arbitrarily small when increasing population subdivision. Recall however that we need to be in the rare mutation regime for our effect to be important. With a mutation probability per nucleotide and per generation of $10^{-10}$ [68], assuming 10 possible mutations for the development of resistance to a given drug, gives $\mu =10^{-9}$. Then, for bacteria dividing once per hour, we find $\langle t_{af\;F}\rangle =10^{8}$ h for *D* = 10, but $\langle t_{af\;F}\rangle =10^{3}$ h for $D=10^{6}$. In the latter case, we would be in the rare mutation regime for deme sizes up to a few hundred bacteria.

Host-associated bacterial populations are often fragmented into numerous small demes, for instance in intestinal crypts, lymph nodes, or skin pores. First, the gut features a rich microbiota which can be a reservoir of resistant bacteria in humans and animals. Intestinal crypts are glandular structures located at the base of the gut lining. The mouse intestine comprises approximately $D=10^{5}$ crypts [69], each harboring about 100 to 400 bacteria [70,71], and the human intestine comprises up to $D=10^{7}$ crypts [72]. Second, lymph nodes may act as reservoirs of bacteria, especially as some antibiotics poorly permeate them [73]. A mouse possesses about *D* = 20 lymph nodes [74], and each mesenteric lymph node holds $10^{3}$ to $10^{5}$ bacteria [75]. Finally, a human face features roughly $D=2\times 10^{4}$ skin pores [76], and a typical skin pore contains about $5\;\times \;10^{4}$ *Cutibacterium acnes* bacteria [77,78]. These orders of magnitude (detailed in S1 Appendix Sect 6) show that the effect of spatial structure on rescue by resistance that we evidenced here may be relevant in such real-world structured populations.

In this work, we focused on a perfect biostatic drug that prevents any division of sensitive bacteria. However, our main findings generalize to biocidal drugs that increase the death rate of sensitive bacteria, or to drugs which combine both modes of actions. Indeed, what happens before the addition of drug, in particular the local fixation of resistant mutants, is not impacted by the mode of action of the drug. Thus, the effect of spatial structure we evidenced here holds independently of this. The difference between the biostatic and the biocidal case lies in the decay of the sensitive bacteria once drug is added. In the biocidal case, sensitive microbes may still divide during this phase, and new resistant mutants may then appear [54,79]. This is the case both in well-mixed and in structured populations, with only minor differences between them, due to the stochasticity of extinction. Thus, our main findings are robust to the mode of action of the drug. Beyond modes of action, it would be interesting to study the impact of spatial structure on multi-step drug resistance evolution [80].

Here, we considered a minimal model of spatial structure, without any environmental heterogeneities. This allowed us to find a generic effect of spatial structure on the survival of a population to antibiotic treatment. It would be very interesting to extend our work to heterogeneous environments, which are known to have a substantial effect on antibiotic resistance evolution [47–49], and on evolutionary rescue in structured populations [50–52].

Some mechanisms of resistance to antibiotics involve the production of a public good. One prominent example is the beta-lactamase enzyme, which degrades beta-lactam antibiotics in the environment. Spatial structure was recently experimentally shown to have an important impact in this case [15]. Note that collective protection via a public good exists in multiple other cases [81–83]. Recently, a model was developed to describe this phenomenon in a well-mixed population [84,85]. Extending this study to the case of structured populations would be very interesting. More generally, coupling these evolutionary questions to ecological interactions, which also have an interesting interplay with spatial structure [86–88], is an exciting perspective.

It would be interesting to test our predictions experimentally. *In vitro* experiments considering bacterial populations with migrations between demes have been performed [37–41], but generally with a constant environment. Investigating the fate of populations upon the addition of drug in these setups would be very interesting. A challenge is that the timescales relevant here are long. However, they can become smaller if the number *D* of demes is increased. Long experiments with large numbers of demes can be achieved using robots for serial passage [37,89,90], and the connection between theory and experiment for spatially structured populations is progressing [91].

## Supporting information

S1 Appendix The Supplementary Appendix comprises derivations of the analytical results presented in the main text, and descriptions of the numerical simulations we performed. It also presents some additional results on different aspects mentioned in the main text.
(PDF)
